# Structures of Phytosterols and Triterpenoids with Potential Anti-Cancer Activity in Bran of Black Non-Glutinous Rice

**DOI:** 10.3390/nu7031672

**Published:** 2015-03-06

**Authors:** Panawan Suttiarporn, Watcharapong Chumpolsri, Sugunya Mahatheeranont, Suwaporn Luangkamin, Somsuda Teepsawang, Vijittra Leardkamolkarn

**Affiliations:** 1 Center of Excellence for Innovation in Chemistry and Department of Chemistry, Faculty of Science, Chiang Mai University, Chiang Mai 50200, Thailand; E-Mails: panawansut@gmail.com (P.S.); watcharapong_chum@hotmail.com (W.C.); 2 Department of Basic Science and Physical Education, Faculty of Science at Si Racha, Kasetsart University, Si Racha Campus, Chonburi 20230, Thailand; E-Mail: lsuwaporn@gmail.com; 3 Department of Anatomy, Faculty of Science, Mahidol University, Bangkok 10400, Thailand; E-Mails: jookkajoon_pt@hotmail.com (S.T.); vijittra.lea@mahidol.ac.th (V.L.)

**Keywords:** rice bran, anti-cancer, gramisterol, lupeol, GC-MS, LC-MS

## Abstract

Structures of some bioactive phytochemicals in bran extract of the black rice cv. Riceberry that had demonstrated anti-cancer activity in leukemic cell line were investigated. After saponification with potassium hydroxide, separation of the unsaponified fraction by reversed-phase high performance liquid chromatography (HPLC) resulted in four sub-fractions that had a certain degree of anti-proliferation against a mouse leukemic cell line (WEHI-3 cell), this being IC_50_ at 24 h ranging between 2.80–467.11 μg/mL. Further purification of the bioactive substances contained in these four sub-fractions was performed by normal-phase HPLC. Structural characterization by gas chromatography-mass spectrometry (GC-MS), liquid chromatography-mass spectrometry (LC-MS) and nuclear magnetic resonance spectroscopy (NMR) resulted in, overall, the structures of seven phytosterols and four triterpenoids. Four phytosterols, 24-methylene-ergosta-5-en-3β-ol, 24-methylene-ergosta-7-en-3β-ol, fucosterol, and gramisterol, along with three triterpenoids, cycloeucalenol, lupenone, and lupeol, were found in the two sub-fractions that showed strong anti-leukemic cell proliferation (IC_50_ = 2.80 and 32.89 μg/mL). The other sterols and triterpenoids were campesterol, stigmasterol, β-sitosterol and 24-methylenecycloartanol. Together with the data from *in vitro* biological analysis, we suggest that gramisterol is a significant anti-cancer lead compound in Riceberry bran extract.

## 1. Introduction

Rice is consumed by over half the world’s population, the majority of which is in Asia. Although white rice is more common, there are many special rice cultivars that contain pigments, such as black, purple, and red, in their bran, pelea, and lemma, and other inside parts, such as pericarp tegment and the aleurone layer. The bran part of these pigmented rice cultivars contains acetylated procyanidin [1], anthocyanins, and other phenolics that have significant free radical scavenging activity [2,3] apart from natural antioxidants, such as tocopherols, tocotrienols [4], oryzanols [5], phytosterols [6], and phenolic compounds [7]. It is believed that consumption of pigmented rice improves human health because of the rice’s antiallergic, antimutagenic, and anticarcinogenic effects [8,9]. Therefore, pigmented rice is becoming a valuable source for food supplements and nutraceuticals.

Thai black rice, cv. Riceberry, has recently been developed to provide optimum nutritional benefit to consumers and to be a supplement for anemic and diabetes mellitus patients since it contains much iron and little glucose. The extract of Riceberry bran has recently been reported by our group to have potential chemopreventive activity against human cancer cell lines, Caco-2, MCF-7, and HL-60 [10]. This extract also has hypoglycemic, hypolipidemic, antioxidant, and anti-inflammation properties [11]. Riceberry bran oil supplement is also noted to be beneficial to oxidative stress and organ histology in streptozotocin-induced diabetic rats fed with a high fat diet [12].

As a great deal of attention has been paid in studies to the structure-activity relationship of phytosterols and triterpenoids in many medicinal plants. Our current studies intended, therefore, to identify the sterols and triterpene alcohols in the unsaponified fraction of the Riceberry extract that possessed anticancer activity, which had been reported previously [10]. In order to accomplish the necessary purification step, both reversed- and normal-phase HPLC were utilized. Additionally, the spectroscopic data useful for structural identification were obtained by NMR with the aid of combined chromatographic-mass spectrometric techniques (GC-MS and LC-MS).

## 2. Materials and Methods

### 2.1. Plant Materials

Thai black non-glutinous rice, cv. Riceberry, was used in this study. This rice was grown in an experimental field in Kasetsart University, Nakorn Pathom province, in central Thailand in 2011. The harvested paddy was dried by modified hot air at a temperature of 40 °C until their moisture content was reduced to approximately 14% by weight. The rice was then stored in a controlled room at 15 °C. On the day of the experiment, the paddy was dehusked and milled in a local milling system (Natrawee Technology, Chachoengsao, Thailand) for 30 s to obtain approximately 10% (w/w) fresh rice bran. The bran was sieved through a 750-lm Endecotts test sieve (Endecotts Ltd., London, UK) prior to further experiments.

### 2.2. Chemicals

Solvents, mainly methanol, dichloromethane, and hexane, used for extraction of chemical components from the Riceberry bran were analytical grade and were obtained from Fluka (Buchs, Switzerland). HPLC grade methanol and hexane and absolute ethanol were purchased from Merck (Darmstadt, Germany). De-ionized water was obtained from a Milli-Q UV-Plus water purification system (Millipore Corp., Billerica, MA, USA).

### 2.3. Extraction and Saponification

One kilogram of the black rice bran was extracted using hexane followed by dichloromethane. The Riceberry bran dichloromethane extract (RBD) was evaporated to dryness yielding 45.9 g of crude extract. The extract was then saponified with 0.5 M ethanolic KOH solution by refluxing at 70 °C for 2 h. After evaporation to dryness, the residue was dissolved in 50 mL of distilled water and partitioned twice with dichloromethane. These two dichloromethane phases were combined and concentrated to give the Riceberry bran dichloromethane extract after saponification (RBDS).

### 2.4. Fractionation and Purification of Sterols and Triterpenoids

Fractionation of the RBDS was done using a HPLC system (Agilent Technologies, Santa Clara, CA, USA). The RBDS was subjected to separation by reversed-phase HPLC on a semi-preparative Vertisep™ C18 column (7.8 mm × 100 mm, 5 μm), at 25 °C with the DAD detector at wavelength 210 nm. Its components were eluted by 100% methanol at a flow rate of 2 mL/min. This yielded four sub-fractions, RBDS1-RBDS4, each of which contained a mixture of phytosterols and triterpenoids. These sub-fractions were subjected to further purification by normal-phase HPLC on a Vertisep™ UPS silica column (4.6 mm × 250 mm, 5 μm) with 1% ethanol in hexane as the mobile phase.

### 2.5. GC-MS and LC-MS Analysis

GC-MS analysis was done with a fused silica AT-5MS capillary column (30 m × 0.25 mm i.d., 0.25 μm film thickness) equipped on a gas chromatography (GC)-Agilent 6890 gas chromatograph-mass spectrometer that had an HP 5973 mass-selective detector (Agilent Technologies, Santa Clara, CA, USA). Its split ratio of injection was 20:1. The injection port temperature was 250 °C. The column temperature program was started at 60 °C upon injection. The temperature was increased at a rate of 3 °C /min to 280 °C and held for 30 min. Purified helium at a flow rate of 1 mL/min was used as the GC carrier gas. The mass spectrometer was operated in electron impact mode: electron energy 70 eV; ion source temperature 230 °C; quadrupole temperature 150 °C; mass range (mass to charge ratio*, m/z*) 29–550; scan rate 6.35 scan/s; EM voltage 1341 V. The GC-MS transfer line was set to 280 °C. Identification of the separated components was performed by matching their mass spectra with reference spectra compiled in the W8N08 and Wiley7n mass spectral libraries (Agilent Technologies, Santa Clara, CA, USA). To determine individual sterol and triterpenoid components in the crude rice bran extract, unsaponified fraction, and its four sub-fractions, a linear calibration curve of β-sitosterol obtained with a correlation coefficient of R^2^ = 0.9998 over the concentration range of 10–400 μg/mL was used. Peak areas of all detected sterols and triterpenoids were measured as the areas under peaks in the GC-MS chromatograms. Concentrations of each component detected were calculated based on equivalence with β-sitosterol.

The liquid chromatography-atmospheric pressure chemical ionization-mass spectrometry (LC-APCI-MS) was operated in positive mode. The optimized atmospheric pressure chemical ionization-mass spectrometry (APCI-MS) parameters were: fragmentor voltage 120 V, capillary voltage 4500 V, drying gas temperature 330 °C, drying gas flow rate 5 L/min, nebulizer pressure 25 psi, and vaporizer temperature 350 °C.

### 2.6. NMR Characterization

Pure sterol and triterpenoid components were characterized by elucidating ^1^H and ^13^C-nuclear magnetic resonance spectra (Bruker AVANCE 400 NMR spectrometer in CDCl_3_). The chemical shifts were in ppm and coupling constants in Hz. Abbreviations used for multiplicity were s = singlet, d = doublet, t = triplet, dt = doublet of triplet, dd = double of doublet and m = multiplet. The dept 135, dept 90, and heteronuclear multiple quantum coherence (HMQC) was done to confirm the carbon multiplicity and carbon positions correlated to protons.

### 2.7. Bioassay of the Rice Bran Sub-Fractions

Cancer blood cells (murine myelomonocytic leukemia cell line; WEHI-3 cells) obtained from ATCC (USA) were plated onto 75 cm^2^ cell culture flasks and grown in RPMI 1640 medium containing 1% penicillin-streptomycin (100 U/mL penicillin and 100 μg/mL streptomycin), 1% glutamine and 10% fetal bovine serum (FBS), at 37 °C under a humidified 5% CO_2_ atmosphere. Two hundred thousand cells were seeded onto 96-well-plates with 100 μL of medium per well and incubated for 24 h to sub-confluence. Following this, the cells were treated with sub-fractions of RBDS dissolved in absolute ethanol at concentrations ranging from 12.5 to 1000 μg/mL for both 24 and 48 h. Absolute ethanol was used to treat cells in the vehicle controls. After incubation, the supernatant was discarded and 100 μL of medium containing 10 μL of 3-(4,5-dimethylthiazol-2-yl)-2,5-diphenyltetrazolium bromide (MTT) solution (5 mg/mL, Sigma, Saint Louis, MO, USA) was added and further incubated at 37 °C for 4 h. The supernatant was replaced with 100 μL of MTT solubilizing solution (10% of triton X-100, 10% of 0.1N HCl, 80% isopropanol). The purple formazan product was measured by spectrophotometer at 570 nm with a reference wavelength at 690 nm. All experiments were performed in triplicate. The percentage of cell viability was statistically analyzed using one-way ANOVA, followed by Tukey *post hoc* test and expressed as mean ± S.E.M. The significant difference between groups was considered at *p* < 0.05.

## 3. Results

### 3.1. Sterols and Triterpenoids Found in Unsaponified Fraction and Sub-Fractions of the Rice Bran Extract

Phytosterol and triterpenoid components in all Riceberry bran extract and fractions, crude dichloromethane (RBD) and the unsaponified dichloromethane fraction (RBDS) and its four sub-fractions (RBDS1-RBDS4), were separated and tentatively identified using a capillary GC-MS. The quantities of these sterols and triterpenoids in the rice bran extract and its fractions are shown in Table 1. Four compounds were examined in the crude dichloromethane: three sterols, β-sitosterol, campesterol, and stigmasterol, and one triterpenoid, 24-methylenecycloartanol. Whereas four sterols, β-sitosterol, campesterol, gramisterol, and stigmasterol, and two triterpinoids, 24-methylenecycloartanol and cycloeucalenol, were identified in the RBDS fraction. The addition of these sterol and triterpenoid components was could be resulted from the elimination of triglyceride and fatty acid matrix by saponification. Another reason was probably due to hydrolysis of ester bond of oryzanols, steryl fatty acyl esters (SE), and hydroxycinnamate steryl esters (HSE).

**Table 1:** Sterol and triterpenoid compositions in the rice bran extract and fractions quantified by GC-MS

Compounds	Yield mg/kg Bran	Percent (%) in Extract	Yield mg/kg Bran	Percent (%) in Fraction	Yield mg/kg Bran	Percent (%) in Fraction
	**RBD**		**RBDS**		**RBDS1**	
**Sterols**						
24-Methylene-ergosta-5-en-3β-ol					298.79	58.13
24-Methylene-ergosta-7-en-3β-ol					197.98	41.88
Fucosterol						
Gramisterol			187.44	9.86		
Campesterol	34.36	0.38	200.31	10.57		
Stigmasterol	39.02	0.41	114.30	5.82		
β-Sitosterol	80.79	0.93	377.26	20.35		
**Triterpenoids**						
Cycloeucalenol			56.43	2.62		
Lupenone						
Lupeol						
24-Methylenecycloartanol	83.92	0.51	830.11	45.38		
	**RBDS2**		**RBDS3**		**RBDS4**	
**Sterols**						
24-Methylene-ergosta-5-en-3β-ol						
24-Methylene-ergosta-7-en-3β-ol						
Fucosterol	398.90	15.80				
Gramisterol	1336.32	56.60				
Campesterol			2939.71	71.15		
Stigmasterol			1198.00	28.85		
β-Sitosterol					3803.12	53.28
**Triterpenoids**						
Cycloeucalenol	184.68	7.75				
Lupenone	83.69	3.12				
Lupeol	96.32	3.34				
24-Methylenecycloartanol					3417.02	46.72

RBDS was fractioned by reversed-phase-HPLC into four fractions, RBDS1, RBDS2, RBDS3, and RBDS4. RBDS1 contained a mixture of two sterols, 24-methylene-ergosta-5-en-3β-ol and 24-methylene-ergosta-7-en-3β-ol, (58.13% and 41.88%, respectively). RBDS2 was derived from the combination of two sterols and three triterpenoids, gramisterol, fucosterol, cycloeucalenol, lupeol, and lupenone, (15.80%, 56.80%, 7.75%, 3.12% and 3.34%, respectively). RBDS3 was the product of two sterols, campesterol and stigmasterol, (71.15% and 28.85%, respectively) while RBDS4 was a mixture of β-sitosterol and a triterpenoid, 24-methylenecycloartanol, (53.28% and 46.72%, respectively). Purification of RBDS4 yielded β-sitosterol and 24-methylenecycloartanol. Five additional structures of sterols and triterpenes, 24-methylene-ergosta-5-en-3β-ol, 24-methylene-ergosta-7-en-3β-ol, fucosterol, lupeol, and lupenone, were confirmed. The most abundant compounds among these rice bran fractions were 24-methylenecycloartanol and β-sitosterol.

### 3.2. Identification and Characterization of the Black Rice Sterol and Triterpenoid Components

Sterols and triterpene alcohols in the RBDS1-RBDS4 sub-fractions of the Riceberry extract were firstly identified by comparing their mass spectra with literature data of pure compounds. Further separation of some components was done by using normal-phase HPLC to obtain pure compounds. Following this, the ^1^H and ^13^C-NMR were done to confirm the carbon multiplicity. The carbon positions were then correlated to protons.

Nine sterol and triterpenoid compounds, 24-methylene-ergosta-5-en-3β-ol, fucosterol, campesterol, stigmasterol, β-sitosterol, cycloeucalenol, lupenone, lupeol, and 24-methylenecycloartanol, were tentatively identified by GC-MS. Two sterols, 24-methylene-ergosta-7-en-3β-ol and gramisterol, were characterized by comparing their ^1^H and ^13^C NMR spectra with literature data. The molecular ion peak of 24-methylene-ergosta-7-en-3β-ol was at *m/z* 398 in its EI mass spectrum and the molecular formula of C_28_H_46_O was investigated for the compound. Gramisterol had an EI spectrum that possessed molecular ion at *m/z* 412 and the molecular formula of C_29_H_48_O. This sterol’s spectrum showed fragment ions at *m/z* 379 [M-CH_3_]^+^, 379 [M-CH_3_-H_2_O]^+^, 328 [M-C_5_H_9_-CH_3_]^+^, 285 [M-side chain-2H]^+^, and 269 [M-side chain-CH_3_-2H]^+^, all of which confirmed its chemical structure.

The molecular weights of all sterols and triterpenoids were also confirmed by their pseudomolecular ions: [M-H_2_O+H]^+^. This confirmation resulted from normal-phase LC-MS operated in APCI mode. It is to be noted that the protonated molecular ions [M+H]^+^ of 24-methylene-ergosta-7-en-3β-ol (*m/z* 399), cycloeucalenol (*m/z* 427), and 24-methylenecycloartanol (*m/z* 441) were very abundant.

**Figure 1:**
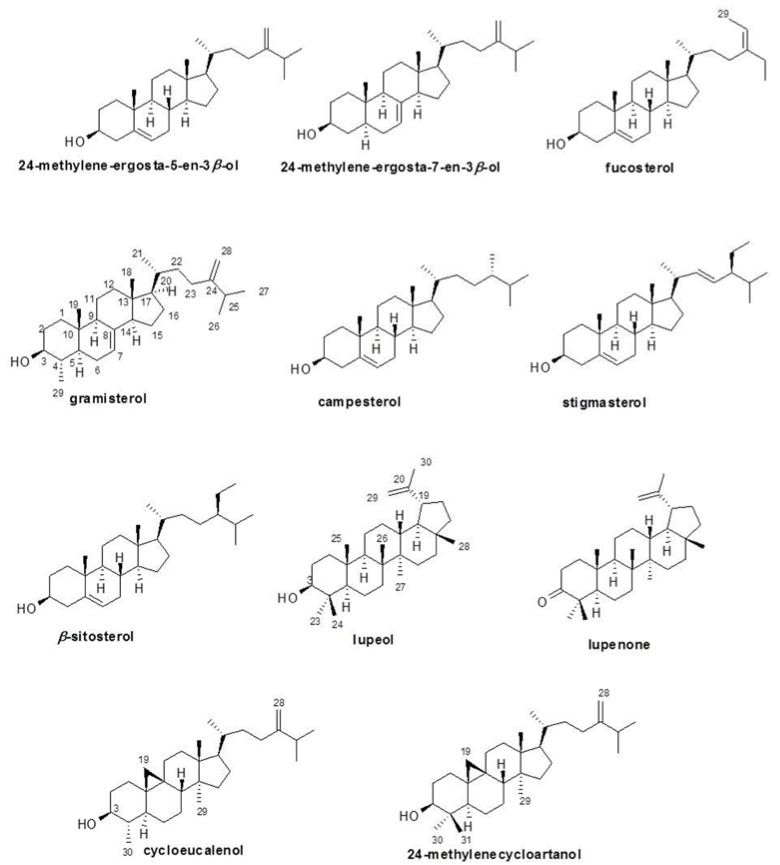
The structures of sterol and triterpenoid compounds obtained from the extract of black rice bran cv. Riceberry.

The ^1^H and ^13^C NMR of 24-methylene-ergosta-5-en-3β-ol also had singlet signals of olefinic methylene protons at δ 4.65 and 4.71 ppm and a doublet signal of olefinic methine proton at δ 5.35 ppm. The carbon signals at δ 105.93, 121.72, and 140.77 ppm supported the two double bonds. The closely related structures of 24-methylene-ergosta-7-en-3β-ol and 24-methylene-ergosta-5-en-3β-ol were differentiated by a double bond in the cyclohexane ring. The different olefinic methine proton appeared as a doublet signal at δ 5.15 ppm and was related to the carbon signal at δ 120.00 ppm. Fucosterol had two double bonds similar to those of 24-methylene-ergosta-5-en-3β-ol but it had a methyl group instead of one olefinic methylene proton at a side chain. Thus, the additional methyl group and olefinic methine proton signals were doublet and quartet at δ 1.57 and 5.18 ppm, respectively. The ^13^C-NMR spectral data of fucosterol showed both carbons at δ 13.05 and 116.46 ppm. Gramisterol had a methyl group instead of a methylene proton of 24-methylene-ergosta-7-en-3β-ol at position four. The additional methyl group proton signal was a doublet at δ 0.95 ppm and was related to the methyl carbon signal at δ 15.15 ppm. Campesterol was related to 24-methylene-ergosta-5-en-3β-ol and had only one double bond in the cyclohexane ring. The doublet signal of olefinic methine proton occurred at δ 5.33 ppm and was related to the carbon signal at δ 121.72 ppm. ^1^H-NMR spectral data of stigmasterol had three olefinic methine proton signals of two double bonds at δ 5.33, 5.15, and 5.00 ppm and they were related to four olefinic carbon signals at δ 121.72, 129.29, 138.32, and 140.77 ppm. The structure of β-sitosterol is similar to stigmasterol but it has a saturated side chain that has only one double bond in the cyclohexane ring. Thus, one olefinic proton doublet signal at δ 5.35 ppm was related to the carbon signal at δ 121.72 ppm. The structure of cycloeucalenol has cyclopropane joined to cyclohexane at positions nine and ten. The methylene protons of cyclopropane occurred as two doublet signals at δ 0.15 and 0.38 ppm. There are two methyl groups in 24-methylenecycloartanol at position four of cyclohexane, while cycloeucalenol has only one methyl group at position four. Thus, seven methyl proton signals at δ 0.81, 0.89, 0.90, 0.97 (2C), 1.02, and 1.04 ppm were shown in ^1^H-NMR spectral data of 24-methylenecycloartanol, which were related to seven carbon signals at δ 14.15, 18.04, 18.32, 19.33, 21.88, 22.01, and 25.24 ppm. The methylene protons of cyclopropane occurred as two doublet signals at δ 0.33 and 0.55 ppm. The olefinic methylene protons occurred as two singlet signals at δ 4.66 and 4.71 ppm and were related to carbon signals at δ 105.94 ppm. The structures and data of these black rice sterols and triterpenoids are summarized in Figure 1 and Table 2 and Table 3.

**Table 2:** Mass spectral data of the identified black rice bran sterols and triterpenoids

Compound	Molecular Formula	Molecular Weight	Major Fragment Ion (*m/z*)	
			LC-APCI-MS	GC-MS
**Sterols**				
24-Methylene-ergosta-5-en-3β-ol	C_28_H_46_O	398	381 [M-H_2_O+H]^+^	398 [M]^+^, 383 [M-CH_3_]^+^ 365 [M-CH_3_-H_2_O]^+^ 314 [M-C_5_H_9_-CH_3_]^+^ 299 [M-C_7_H_13_-2H]^+^ 281 [M-C_7_H_13_-H_2_O-2H]^+^ 271 [M-side chain-2H] ^ +^
24-Methylene-ergosta-7-en-3β-ol	C_28_H_46_O	398	381 [M-H_2_O+H]^+^,399 [M+H]^+^	398 [M]^+^, 383 [M-CH_3_]^+^ 365 [M-CH_3_-H_2_O]^+^ 314 [M-C_5_H_9_-CH_3_]^+^ 299 [M-C_7_H_13_-2H]^+^ 271 [M-side chain-2H]^+^
Fucosterol	C_29_H_48_O	412	395 [M-H_2_O+H]^+^	412 [M]^+^, 397 [M-CH_3_]^+^ 379 [M-CH_3_-H_2_O]^+^ 314 [M-C_6_H_11_-CH_3_]^+^ 299 [M-C_6_H_11_-2CH_3_]^+^ 281 [M-C_6_H_11_-2CH_3_-H_2_O]^+^ 229 [M-side chain-ring D cleavage-CH_3_-2H]^+^ 213 [M-side chain-ring D cleavage-CH_3_-H_2_O]^+^
Gramisterol	C_29_H_48_O	412	395 [M-H_2_O+H]^+^	412 [M] ^+^, 397 [M-CH_3_]^+^ 379 [M-CH_3_-H_2_O]^+^ 328 [M-C_5_H_9_-CH_3_]^+^ 285 [M-side chain-2H] ^+^ 269 [M- side chain -CH_3_-2H]^ +^
Campesterol	C_28_H_48_O	400	383 [M-H_2_O+H]^+^	400 [M]^+^, 385 [M-CH_3_]^+^ 382 [M-H_2_O]^+^ 367 [M-CH_3_-H_2_O]^+^
Stigmasterol	C_29_H_48_O	412	395 [M-H_2_O+H]^+^	412 [M]^+^, 397 [M-CH_3_]^+^ 394 [M-H_2_O]^+^ 369 [M-C_3_H_5_]^+^ 351 [M-C_3_H_5_-H_2_O]^+^ 314 [M-C_7_H_14_]^+^ 271 [M-side chain -2H]^+^ 255 [M-side chain-H_2_O]^+^ 213 [M-side chain-ring D cleavage-H_2_O]^+^
β-Sitosterol	C_29_H_50_O	414	397 [M-H_2_O+H]^+^	414 [M]^+^, 399 [M-CH_3_]^+^ 396 [M-H_2_O]^+^ 381 [M-CH_3_-H_2_O]^+^ 329 [M-C_6_H_13_]^+^ 303 [M-C_7_H_11_O]^+^ 273 [M-side chain]^+^ 255 [M-side chain -H_2_O]^+^ 231 [M-side chain-ring D cleavage-CH_3_]^+^ 213 [M-side chain-ring D cleavage-CH_3_-H_2_O]^+^		
**Triterpenoids**				
Cycloeucalenol	C_30_H_50_O	426	409 [M-H_2_O+H]^+^, 427 [M+H]^+^	426 [M]^+^, 411 [M-CH_3_]^+^ 408 [M-H_2_O]^+^ 393 [M-CH_3_-H_2_O]^+^ 353 [M-C_3_H_7_-2CH_3_-H_2_O]^+^ 300 [M-C_7_H_13_-CH_2_-CH_3_]^+^		
Lupenone	C_30_H_48_O	424	ND (minor compound)	424 [M]^+^, 409 [M-CH_3_]^+^ 381 [M-side chain -2H]^+^ 368 [M-side chain-CH_3_]^+^ 342 [M-side chain-ring E cleavage]^+^ 313 [M-C_7_H_11_O]^+^		
Lupeol	C_30_H_50_O	426	ND (minor compound)	426 [M]^+^, 411 [M-CH_3_]^+^ 393 [M-CH_3_-H_2_O]^+^ 383 [M-side chain-2H]^+^ 370 [M-side chain -CH_3_]^+^ 329 [M-side chain-ring E clevage-CH_3_]^+^		
24-Methylenecycloartanol	C_31_H_52_O	440	423 [M-H_2_O+H]^+^,441 [M+H]^+^	440 [M]^+^, 425 [M-CH_3_]^+^ 422 [M-H_2_O]^+^ 407 [M-CH_3_-H_2_O]^+^ 397 [M-C_3_H_7_]^+^ 379 [M-C_3_H_7_-H_2_O]^+^ 315 [M-C_9_H_17_]^+^ 300 [M-C_9_H_17_-CH_3_]^+^ 285 [M-C_9_H_17_-2CH_3_]^+^ 203 [M-C_9_H_17_-5CH_3_]^+^		

**Table 3:** NMR data of the identified black rice bran sterols and triterpenoids

Compound	Selected ^1^H-NMR Data δ (Multiplicity/Hz)	Selected ^13^C-NMR Data δ
**Sterols**		
24-Methylene-ergosta-5-en-3β-ol	0.68 (*s*, H_18_), 0.95 (*d*, 6.6, H_21_), 1.00 (*s*, H_19_), 1.02, 1.03 (*d*, 6.8, H_26_, H_27_), 3.52 (*m*, H_3_), 4.65 (*s*, H_28_), 4.71 (*s*, H_28_), 5.35 (*d*, 4.9, H_6_)	11.86 (C_18_), 18.71 (C_21_), 19.40 (C_19_), 21.87, 22.00 (C_26_, C_27_), 71.82 (C_3_), 105.93 (C_28_), 121.72 (C_6_), 140.77 (C_5_)
24-Methylene-ergosta-7-en-3β-ol	0.54 (*s*, H_18_), 0.80 (*s*, H_19_), 0.94 (*d*, 6.6, H_21_), 1.02, 1.03 (*d*, 6.8, H_26_, H_27_), 3.62 (*m*, H_3_), 4.65 (*s*, H_28_), 4.71 (*s*, H_28_), 5.15 (*d*, 2.3, H_7_)	11.86 (C_18_), 13.04 (C_19_), 18.84 (C_21_), 21.87, 22.00 (C_26_, C_27_), 71.08 (C_3_), 105.93 (C_28_), 120.00 (C_7_), 139.57 (C_8_)
Fucosterol	0.68 (*s,* H_18_), 0.98 (*d,* 6.4, H_21_), 1.01, 1.02 (*d,* 6.8, H_26_, H_27_), 1.00 (*s,* H_19_), 1.57(*d,* 7.2, H_29_), 3.53 (*m*, H_3_), 5.18 (*q*, 6.7, H_28_), 5.35 (*d,* 5.2, H_6_).	11.85 (C_18_), 13.05 (C_29_), 18.81 (C_21_), 19.40 (C_19_), 21.87 (C_27_), 22.01 (C_26_), 71.82 (C_3_), 116.46 (C_28_), 121.72 (C_6_), 140.77 (C_5_), 146.80 (C_24_).
Gramisterol	0.54 (*s*, H_18_), 0.79 (*s,* H_19_), 0.89 (*d,* 6.5, H_21_), 0.95 (*d,* 6.4, H_29_), 1.01, 1.02 (*d,* 6.8, H_26_, H_27_), 3.11 (*dt*, 4.3, 10.9, H_3_), 4.65 (*s*, H_28_), 4.71 (*s*, H_28_), 5.12 (*d*, 3.9, H_7_).	11.85 (C_18_), 14.14 (C_19_), 15.15 (C_29_), 18.84 (C_21_), 21.37 (C_11_), 21.87 (C_27_), 22.01 (C_26_), 76.23 (C_3_), 105.95 (C_28_), 117.51 (C_7_), 139.10 (C_8_), 156.87 (C_24_).
Campesterol	0.67 (*s*, H_18_), 0.77 (*d,* 6.6, H_28_), 0.79 (*d,* 6.8, H_27_), 0.84 (*d,* 6.8, H_26_), 0.90 (*d,* 6.6, H_21_), 1.00 (*s,* H_19_), 3.51 (*m*, H_3_), 5.33 (*d,* 5.2, H_6_).	11.87 (C_18_), 15.38 (C_28_), 18.26 (C_27_), 18.71 (C_21_), 19.40 (C_19_), 20.21 (C_26_), 71.81 (C_3_), 121.72 (C_6_), 140.77 (C_5_).
Stigmasterol	0.69 (*s*, H_18_), 0.76 (*d,* 7.8, H_29_), 0.80 (*d,* 6.8, H_27_), 0.85 (*d,* 6.8, H_26_), 1.00 (*s,* H_19_), 1.01 (*d,* 6.6, H_21_), 3.51 (*m*, H_3_), 5.00, 5.15 (*dd*, 8.6, 5.2, H_22_, H_23_), 5.33 (*d,* 5.2, H_6_).	12.05 (C_18_), 12.25 (C_27_), 15.15 (C_29_), 18.90 (C_21_), 19.40 (C_19_), 20.52(C_26_), 71.81 (C_3_), 121.72 (C_6_), 129.29, 138.32 (C_22_, C_23_), 140.77 (C_5_).
β-Sitosterol	0.67 (*s*, H_18_), 0.80, 0.82 (*d,* 6.8, H_26_, H_27_), 0.84 (*d,* 7.8, H_29_), 0.92 (*d,* 6.6, H_21_), 1.00 (*s,* H_19_), 3.52 (*m*, H_3_), 5.35 (*d,* 5.2, H_6_).	11.87 (C_18_), 11.99 (C_29_), 18.79 (C_21_), 19.05, 19.83 (C_26_, C_27_), 19.41 (C_19_), 70.79 (C_3_), 121.72 (C_6_), 140.77 (C_5_).
**Triterpenoids**		
Cycloeucalenol	0.15 (*d*, H_19_), 0.38 (*d*, H_19_), 3.20 (*m*, H_3_), 4.64 (*s*, H_28_), 4.73 (*s*, H_28_).	ND (minor compound)
Lupenone	4.57 (*m*, H_29_), 4.68 (*d*, 2.2, H_29_)	ND (minor compound)
Lupeol	3.20 (m, H_3_), 4.57 (*m*, H_29_), 4.68 (*d*, 2.2, H_29_)	ND (minor compound)
24-Methylenecycloartanol	0.33 (*d*, 4.2, H_19_), 0.55 (*d*, 4.2, H_19_), 0.81 (s, H_18_), 0.89 (d, 4.3, H_21_), 0.90 (s, H_29_), 0.97 (s, H_30_, H_31_), 1.02 (d, 2.2, H_26_), 1.04 (d, 2.2, H_27_), 3.28 (*dd*, 4.3, 11.1, H_3_), 4.66 (*s*, H_28_), 4.71 (*s*, H_28_).	14.15, 18.04, 18.32, 19.33, 21.88, 22.01, 25.24 (7Me), 29.72 (C_19_), 78.85 (C_3_), 105.94 (C_28_), 156.92 (C_24_).

### 3.3. Anti-Proliferation (MTT) Activity

**Figure 2:**
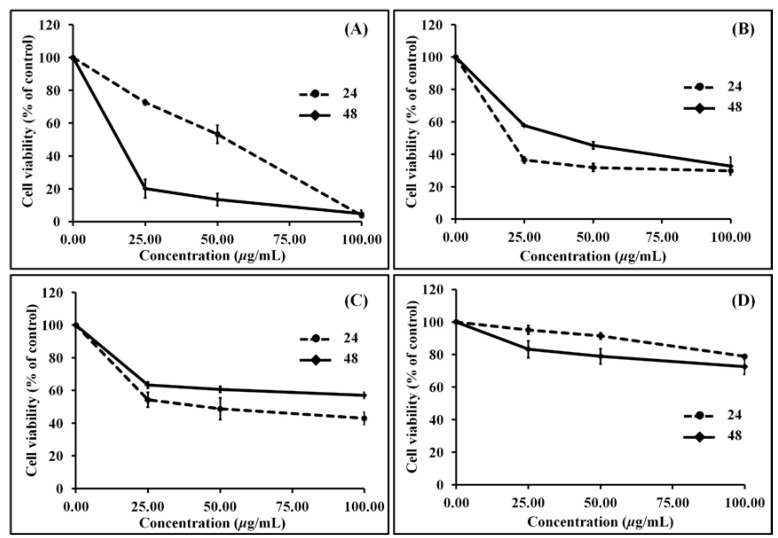
Effect of (**A**) RBDS1; (**B**) RBDS2; (**C**) RBDS3; and (**D**) RBDS4 on cell viability analyzed using MTT assay.

All four sub-fractions of the RBDS showed time-dependent and concentration-dependent anti-proliferation against mouse leukemic cell line (WEHI-3 cells) (Figure 2). The *in vitro* effectiveness of the RBDS1-4 sub-fractions against this cancer cell is indicated by the concentrations that inhibited 50% of cell growth (IC_50_) shown in Table 4. The IC_50_ of these RBDS sub-fractions determined at 24 h ranged from 2.80 to 467.11 μg/mL. RBDS1 and RBDS2 sub-fractions exhibited stronger anti-proliferation of cancer cell than did sub-fractions RBDS3 and RBDS4.

**Table 4:** The 50% inhibitory concentration (IC_50_) of the fractions on WEHI-3 cancer cells

Extracts	IC_50_ WEHI-3 (μg/mL)	
	24 h	48 h
RBDS1	32.89	3.32
RBDS2	2.80	36.30
RBDS3	47.59	376.87
RBDS4	467.11	615.24

## 4. Discussion

The RBD crude extract analyzed by GC-MS gave the detection of only four major phytosterols; campesterol, stigmasterol, β-sitosterol, and 24-methylenecycloartanol, which was due to the high complexity of sample matrices. These four phytosterols, along with gramisterol and cycloeucalenol, were also found in RBDS. The higher numbers of free sterols and triterpene alcohols in the RBDS indicate the important role of these phytochemicals in enabling higher biological performance. However, the relatively highest amount of 24-methylenecycloartanol contained in the RBDS only partially played a role in anti-proliferation of WEHI-3 cells. This was confirmed by approximately the same amount of 24-methylenecycloartanol in RBDS4 sub-fraction having weaker anti-proliferation than that of the other RBDS sub-fractions. Previously, 24-methylenecycloartanol had shown many biological activities, such as arousal effect [13,14], strongly active against 12-O-tetradecanoylphorbol-13-acetate (TPA)-induced inflammation [15], and cytotoxicity against HeLa as well as WI-38 and Mel-43 cancer cells [16]. β-Sitosterol, the second abundant compound in RBDS presented as a free sterol, has several known biological activities: reduction of blood cholesterol [17], anti-inflammation [15], cytotoxic against Hela, Caski, MCF-7, and A546 cancer cells [18,19,20], and anti-diabetic and anti-peroxidation [21]. This phytosterol was found to partially play a role in anti-WEHI-3 cell proliferation because it exerted weaker anti-proliferation at higher concentration in the RBDS4 sub-fraction with 24-methylenecycloartanol compared to the other sub-fractions.

The other compounds that promoted WEHI-3 cell anti-proliferation activity were gramisterol, campesterol, stigmasterol, and cycloeucalenol. Campesterol and stigmasterol had been probed in rice bran oil in the form of free alcohol and steryl ferrulate, which exhibited anti-inflammatory effect and cytotoxic against some cancer cell lines [15,16]. Both compounds occurred in RBDS3 sub-fraction, which presented relatively lower anti-proliferation against WEHI-3 cell. A triterpenoid, cycloeucalenol, occurred in a small amount and, thus, may play significant role in the RBDS or in the RBDS2 sub-fraction.

RBDS1 sub-fraction had the second most active anti-WEHI-3 cancer cell proliferation, presumably because of its 24-methylene-ergosta-5-en-3β-ol and 24-methylene-ergosta-7-en-3β-ol sterols. Both compounds had never been isolated or characterized from rice bran due to their relatively small amounts. Therefore, finding these rice bran sterols and their biological effect on anti-tumor activity is novel. Mostly, edible rice bran contains 24-methylenecholesterol as a free sterol or *trans*- and *cis*-ferulate that had anti-inflammatory activity [22]. The 24-methylene-ergosta-7-en-3β-ol compound had been reported in culms and leaves of traditional African rice, *Oryza glaberimma*, but it had never been shown to benefit human health [23].

In RBDS2 sub-fraction, gramisterol (24-methylenelophenol) was more abundant than any of the other sterols. Therefore, it probably is the most active anti-tumor sterol against WEHI-3 cells and its effect agrees with a previously reported finding [24]. Gramisterol in the form of free alcohol and their *trans*- and *cis*-ferulate has an anti-inflammatory effect [15]. The other minor components in the RBDS2 sub-fraction were less involved in anti-WEHI-3 cancer cell proliferation. Fucosterol was previously detected as a major component in rice bran oil [25,26]. Its known biological activities include anti-histaminic, anti-cholinergic, anti-viral [27], and hepatoprotective activities in rats [28]. Cycloeucalenol has been reported in the form of cycloeucalenol *trans*-ferulate in rice germ and is also found in *Tinospora cordifolia* or Guduchi. Its biological effects include cardiotonic [29] and anti-inflammatory activity [15]. The detection of lupenone and lupeol in rice bran has never been reported, although both have been identified in various therapeutic plants. Therefore, this is the first finding of these two triterpenoids and their potential anti-cancer cell activities. Lupenone and lupeol helped to promote the inhibition of protein tyrosine phosphatase 1B (PTP1B). This significantly decreased insulin activity in diabetes and treated obesity [30]. Lupeol was reported to have anti-inflammatory, anti-tumor, anti-protozoal, and anti-microbial activities. It is also known as a cardio-protective agent [31].

RBDS3 and RBDS 4 had less WEHI-3 anti-proliferation activity than RBDS1 and RBDS2 since they contained compounds that possessed several biological activities and may only partially participate in cancer inhibition.

Phytosterols from plant food-based diet, such as vegetables, cereals, and nuts are bioavailable. This diet is the main source of phytosterols for humans and the intake of phytosterols in human depends on the amount and type of plant products consumed. A natural diet rich in phytosterols is likely to provide around 1000 mg of phytosterols per day [32]. The consumption of naturally occurring phytosterols has been estimated to range from 150 to 450 mg per day [33]. The high amount of phytospterols intake is from vegetarian diet; dietary patterns that are consistent with a reduction of breast cancer risk [34].

Due to the structural differences between phytosterols and cholesterol, phytosterols are less absorbed in animals or humans. Estimates of the bioavailability of dietary sterols have been approximated from controlled intestinal perfusion studies with healthy normal men. Under these experimental settings, sterol bioavailability averaged 33% for cholesterol, 10% for campesterol, 5% for stigmasterol, and 4% for β-sitosterol [35]. The vast majority of the phytosterols ingested remain in the gastrointestinal (GI) tract. In healthy humans, with daily phytosterol intakes of 150 mg/day, only 0.3 mg/dL phytosterols are found in the blood. However, this blood phytosterol concentration is sufficient to reach the anti-leukemic cell proliferation effect of the active black rice bran sub-fraction (IC_50_ 2.80 μg/mL). In order to increase concentration levels of phytosterols in blood, the phytosterols can be extracted from certain plant foods and added in higher doses to various other foods or dietary supplement products. The good understanding of the chemistry and composition of phytochemicals in certain plant food, such as black rice, and especially of the compounds that contribute to its important bioactivity is considered a prerequisite for the works on future functional food development efforts using free phytosterols.

## 5. Conclusions

The relative amounts of seven sterols and four triterpenoids were determined in the unsaponified fraction of the RBD extract of black rice bran cv. Riceberry. The structure of these sterols and triterpenoids were confirmed using GC-MS, LC-MS, ^1^H-NMR, and ^13^C-NMR techniques. Sterols; 24-methylene-ergosta-7-en-3β-ol, and triterpenoids, lupenone, and lupeol were noted for the first time in rice bran extracts. β-Sitosterol was found by GC-MS as the major component, followed by 24-methylenecycloartanol, which was in a significant amount in oryzanols. RBDS2 sub-fraction of rice bran extract that contains significant amount of gramisterol, along with other minor components; fucosterol, cycloeucalenol, lupenone and lupeol, efficiently reduces WEHI-3 cancer cell proliferation. This study showed that gramisterol plays a significant role in anti-leukemic cancer cell. Therefore, it is interesting to explore the biological activities of gramisterol in animal models since it has potential human benefits in terms of anti-cancer drug development.
